# Direct evidence of secondary reconnection inside filamentary currents of magnetic flux ropes during magnetic reconnection

**DOI:** 10.1038/s41467-020-17803-3

**Published:** 2020-08-07

**Authors:** Shimou Wang, Rongsheng Wang, Quanming Lu, Huishan Fu, Shui Wang

**Affiliations:** 1 CAS Key Laboratory of Geospace Environment, Department of Geophysics and Planetary Science, University of Science and Technology of China, Hefei, 230026 China; 2 CAS Center for Excellence in Comparative Planetology, Hefei, China; 3 Anhui Mengcheng Geophysics National Observation and Research Station, University of Science and Technology of China, Mengcheng, 233500 Anhui China; 4 School of Space and Environment, Beihang University, Beijing, China

**Keywords:** Planetary science, Magnetospheric physics

## Abstract

Magnetic reconnection is a fundamental plasma process, by which magnetic energy is explosively released in the current sheet to energize charged particles and to create bi-directional Alfvénic plasma jets. Numerical simulations predicted that evolution of the reconnecting current sheet is dominated by formation and interaction of magnetic flux ropes, which finally leads to turbulence. Accordingly, most volume of the reconnecting current sheet is occupied by the ropes, and energy dissipation occurs via multiple relevant mechanisms, e.g., the parallel electric field, the rope coalescence and the rope contraction. As an essential element of the reconnecting current sheet, however, how these ropes evolve has been elusive. Here, we present direct evidence of secondary reconnection in the filamentary currents within the ropes. The observations indicate that secondary reconnection can make a significant contribution to energy conversion in the kinetic scale during turbulent reconnection.

## Abstract (web summary)

Magnetic reconnection is a fundamental plasma process of magnetic energy conversion to kinetic energy. Here, the authors show direct evidence of secondary reconnection in the filamentary currents within the flux ropes indicating a significant contribution to energy conversion in the kinetic scale during turbulent reconnection.

## Introduction

A macroscale current sheet is one necessary condition for occurrence of magnetic reconnection. When the current sheet thins to ion-scale, it becomes unstable to produce a train of ion-scale magnetic flux ropes characterized by the helical magnetic structures^1–4^. The roles of these flux ropes in reconnection have been extensively investigated, e.g., energizing electrons^1,5–9^, realizing fast reconnection^10,11^, mixing plasma at two sides of current sheet^12,13^, and transferring magnetic fluxes^14,15^. In this process, magnetic free energy is injected at large spatial scale, then transferred from the large scale into the small scale and dissipated at the kinetic scale. This kind of cross-scale energy cascade has been verified^16,17^. The current density within the flux rope was previously derived from magnetic field via Ampere’s law and exhibits a singular compact layer, primarily along the axis^17^. By the accurate plasma moment data in high time resolution recently achieved by magnetospheric multiscale (MMS) mission^18^, the researchers found that the singular compact current layer can be composed of many filamentary electron currents^19,20^, and the filamentary currents were directed at any direction other than only along the rope axis^20^. It suggests that the free energy is transferred into the much-smaller filamentary currents inside the ion-scale flux ropes. How this energy transfer actually occurs and how this energy is finally dissipated inside the flux ropes remain issues.

Here we show identification of series of varied magnetic field pulses inside the flux ropes downstream of a primary reconnection site at the magnetopause. These pulses represent small-scale flux rope-like structures and, within these pulses, a few filamentary currents are observed. Secondary reconnection is detected inside the filamentary currents of the pulses.

## Results

### Overview of the reconnection event

**Fig. 1:**
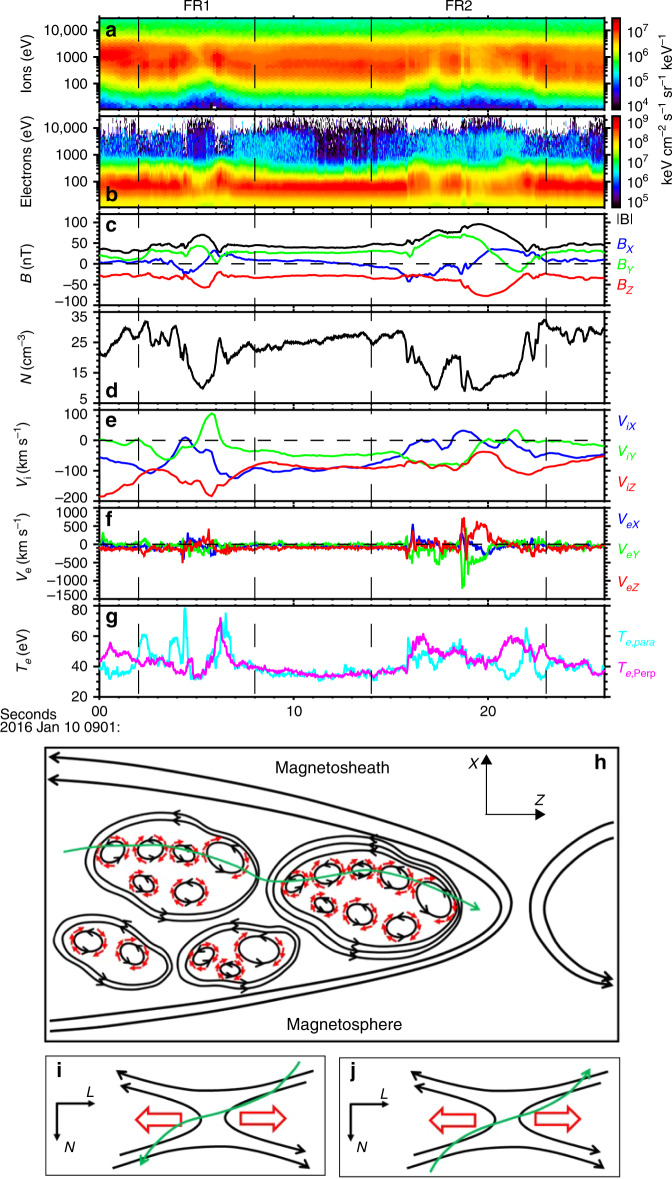
Overview of MMS3 observations. The data are presented in the geocentric solar magnetospheric (GSM) coordinates. The vertical dashed lines mark the leading and trailing edges of the flux ropes. **a**, **b** The ion and electron energy-time spectrograms of differential energy fluxes (color scale, in units of keV cm^−2^ s^−1^ sr^−1^ keV^−1^). **c** The magnitude and components of the magnetic field. **d** Electron density. **e** Ion velocity. **f** Electron velocity. **g** Electron temperature. **h** Schematic showing the varied magnetic field pulses and electron vortices in magnetic flux ropes. Black lines with arrows represent magnetic field lines, red arrows represent electron flows, and green curve with an arrow represents MMS trajectory. **i**, **j** A simplified schematic of reconnection in varied magnetic field pulses. The oppositely directed electron outflows were observed.

Figure 1 shows an overview of the MMS observations dawn-side of Earth’s magnetopause, in the geocentric solar magnetospheric (GSM) coordinates, during 09:01:00–09:01:26 UT on 10 Jan 2016. In this time interval, the spacecraft was located in the magnetopause boundary layer characterized by the southward magnetic field component (Fig. 1c), dominant low-energy (<200 eV) electron population and subordinate high-energy (>1 keV) electron population (Fig. 1b). The continuous southward ion flows were observed in this interval and the average speed was about −100 km s^−1^ (Fig. 1e), ~0.8 *v*_A_, where *v*_A_ was the local Alfvén speed based on *B*_*z*_ ≈ 30 nT and *N* ≈ 25 cm^−3^. It suggests that a primary reconnection event was occurring north of the spacecraft^21^. The MMS spacecraft detected many magnetic flux ropes inside the ion diffusion region^21^, closely analogous to the previous observations in the magnetotail^17^. Here, we focus on the two flux ropes with the longest duration since the microphysics within the flux ropes can be analyzed in detail.

The two flux ropes, named as FR1 and FR2, are characterized by the bipolar *B*_x_ signatures accompanied with the enhancements of magnetic field magnitude and *B*_y_ near the reversal points of *B*_x_ (Fig. 1c), and were bounded by the vertical dashed lines in Fig. 1. The boundary was determined as the location where the magnetic field strength varied from and returned to the ambient value (black curve in Fig. 1c). By the time delay of *B*_x_ = 0 between the four satellites, the timing method^22^ was used to estimate their velocities and the speeds of the two flux ropes were ~119 and ~72 km s^−1^, respectively. The durations of FR1 and FR2 were ~6.0 s (09:01:02.0–09:01:08.0 UT) and ~9.0 s (09:01:14.0–09:01:23.0 UT). Thus, the radii of the two flux ropes were estimated to be ~357.0 km (~7.8 *d*_i_) and ~324.0 km (~7.1 *d*_i_) respectively, where *d*_i_ was ion inertial length, *d*_i_ = 45.6 km based on *N* ≈ 25 cm^−3^.

Inside the two flux ropes, the electron density was largely depressed and had the minimum near the centers (Fig. 1d), with some localized peaks. The electron temperature increased inside the flux ropes and fluctuated strongly (Fig. 1g). Remarkably, a series of electron flow spikes were observed and the speed occasionally exceeded ~1000 km s^−1^ (Fig. 1f), much larger than the ion flow speed (≤200 km s^−1^, Fig. 1e). These electron flow spikes referred to the thin electron current layers inside the flux ropes and would be explored later.

### Varied magnetic field pulses inside flux ropes

**Fig. 2:**
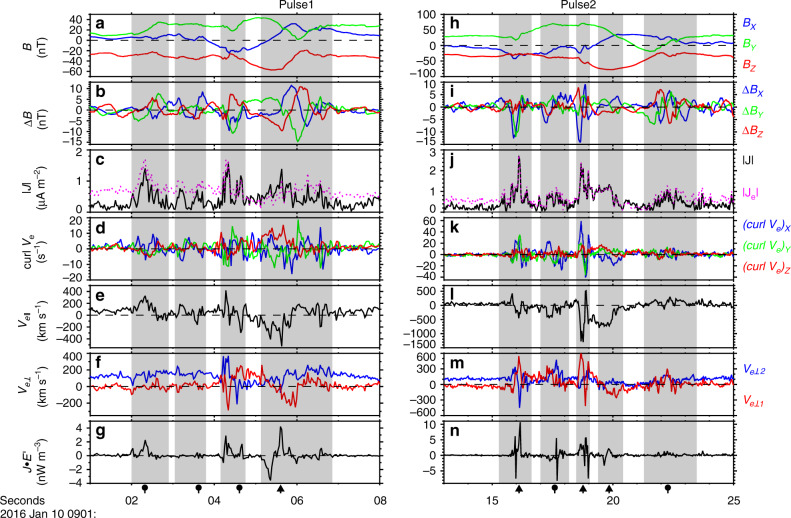
Enlarged view of two flux ropes. The two ropes correspond to the left and right columns. The vertical shadows mark the varied magnetic field pulses. Different black arrows below the vertical shadows represent two kinds of varied magnetic field pulses with different signatures. **a** Three components of the magnetic field in GSM coordinates. **b** The perturbation in magnetic field *B* with the 1-s average data removed. **c** The total (black trace) and electron (pink trace) current density intensity. The total current density intensity $|{{J}}|\,=\,{{e}}N|{{V}}_{\rm{i}}\,-\,{{V}}_{\rm{e}}|$, the electron current density intensity $|{{J}}_{{e}}|\,=\,{{e}}N| - {{V}}_{{e}}|$, where *e* is the elementary charge, *N* is the electron number density, and *V*_i_ and *V*_e_ are the ion and electron velocity. **d** The curl of electron velocity. **e** The parallel electron velocity. **f** The two perpendicular components of the electron velocity, ⊥1 along (*v*_e_ × *b*) and ⊥2 along *b* × (*v*_e_ × *b*), where *b* and *v*_e_ are unit vectors of the background magnetic field and the electron flow, respectively, calculated with the background average value outside the flux ropes. Here, *b* = (0.188, 0.679, −0.709) and *v*_e_ = (−0.500, −0.124, −0.857) determined from interval 09:01:10.0–09:01:11.5 UT. **g***J* • *E*′ = *J* • (*E* + *V*_e_ × *B*). **h**–**n** The data of the rope FR2 are displayed in the same form and *b* = (0.176, 0.657, −0.733) and *v*_e_ = (−0.502, −0.334, −0.798) for FR2 determined from interval 09:01:24.5–09:01:26.0 UT.

The features of the magnetic field and electron data inside the two flux ropes are enlarged in Fig. 2a–n. The signatures of magnetic flux ropes are evident from Fig. 2a, h. Figure 2b, i show the varied magnetic field in the three directions with the 1-s average data removed. The magnetic field fluctuations were strong inside the flux ropes and a train of the varied magnetic field pulses were observed in both flux ropes, marked by the vertical shadows. Figure 2c, j display the total (black trace) and electron (pink trace) current density intensities. A number of the current density peaks were observed within both flux ropes, i.e., the filamentary currents^19,20^. The total current density was nearly equal to the electron current density at most of peaks within the FR1 (Fig. 2c) and almost all peaks inside the FR2 (Fig. 2j). It appears that most of the filamentary currents were primarily carried by the electrons. These filamentary currents were all located within the varied magnetic field pulses and were the reason for the magnetic pulses within the ropes. At least two components of the varied magnetic field reversed inside each pulse (Fig. 2b, i). On average, Δ*B* was up to 10 nT, ~ 0.2|*B*|. It means that the varied magnetic field pulses signify a kind of helical magnetic topology, similar to the magnetic flux ropes in small-scale, which was very evident even in the three magnetic field components, in the pulses at ~09:01:06 UT (dubbed Pulse1 at the top of Fig. 2) within FR1 and at ~09:01:19 UT (Pulse2) within FR2. It was illustrated in Fig. 1h.

Figure 2d, k show the electron flow vorticity calculated by the flow measurements at four satellites. All three components of electron vorticity were significant in each varied magnetic field pulse. It means that the electron flows were very complicated inside the pulses and there were the electron vortices in the pulses. In order to figure out the electron vortices, the electron flows were transferred into the field-aligned coordinates (//, ⊥1, ⊥2), where // along *b* direction, ⊥1 along (*v*_e_ × *b*) direction, and ⊥2 along *b* × (*v*_e_ × *b*) direction, where *b* and *v*_e_ are the unit vectors of the background magnetic field and the background electron flow, respectively, calculated with the background average value outside the flux ropes. The parallel electron flows are displayed in Fig. 2e, l while the two perpendicular components are shown in Fig. 2f, m. The parallel electron flow reversed inside all of the varied magnetic field pulses and, sometimes, changed sign multiple times in a single pulse, e.g. at ~09:01:04.3 UT, ~09:01:06.0 UT, ~09:01:16.0 UT, ~09:01:19.0 UT. The perpendicular component *V*_e⊥1_ reversed as well within each varied magnetic field pulse (red curves in Fig. 2f, m). In the pulses with multiple reversals of *V*_e||_ listed above, the electron flow in the *V*_e⊥2_ also changed sign. It appears that the electron flow reversed at least in two directions inside all of the pulses. During some of the pulses, all three components of the electron flows changed direction. Thus, it is concluded that there were electron vortices inside the varied magnetic field pulses. Sometimes, the electron vortices were three-dimension eddies.

**Fig. 3:**
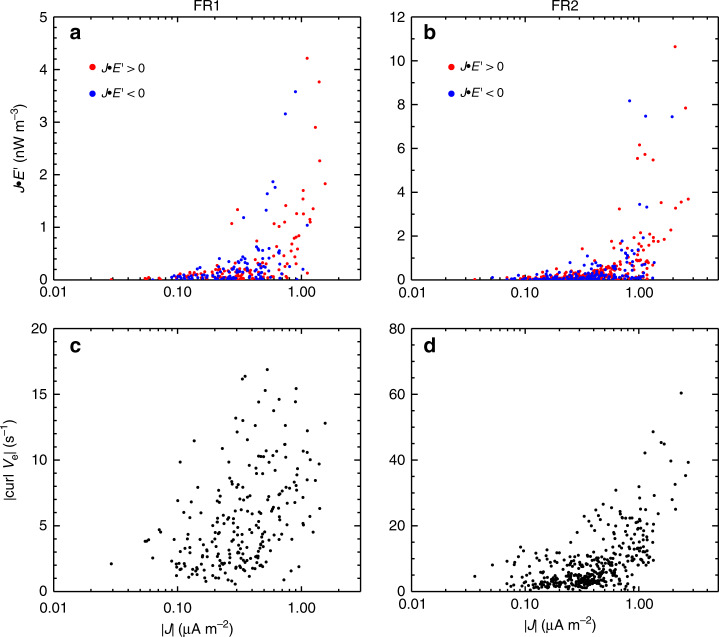
The scatter plots of *J* • *E*′, |∇ × ***V***_**e**_| and |***J***|. **a**, **b** The scatter plot of *J* • *E*′ and current density intensity |*J*| inside FR1 and FR2. The red (blue) dots represent the positive (negative) *J* • *E*′ data points. **c**, **d** The scatter plot of intensity of the electron velocity curl and |*J*| inside FR1 and FR2.

Figure 2g, n show *J* • *E*′, where *E*′ = *E* + *V*_e_ × *B*, and *J* • *E*′ denotes the energy conversion from electromagnetic field to plasmas in the electron rest frame^23^. *J* • *E*′ had a few peaks and valleys inside most of the varied magnetic field pulses, and the *J* • *E*′ peaks and valleys persisted for a short while (<0.5 s). In other words, the strong energy conversion occurred in the extremely localized area within the pulses. In order to figure out where the energy conversion happened, the scatter plot of *J* • *E*′ and |*J*| inside FR1 and FR2 are, respectively, displayed in Fig. 3a, b. It is clear that the energy conversion mainly occurred in the region with strong current density. The most intense energy conversion (|*J* • *E*′| > 2 nW m^−3^) only appeared at |*J*| ≥ 0.8 µA m^−2^. Such a relation has been demonstrated in the plasma turbulence using numerical simulations^24^. Furthermore, the total amount of all *J* • *E*′ data points was 16.2 nW m^−3^ inside FR1 and 65.7 nW m^−3^ inside FR2. This means that magnetic free energy was converted into the plasma energy inside both big flux ropes. The relation between the electron vorticity and the current density intensity was shown in Fig. 3c, d. Basically, the electron vorticity was intensified as increase of the total current density. The relation between the electron vorticity and the filamentary currents was observed also in the reconnection exhaust region^25^.

### Reconnection in the varied magnetic field pulses

**Fig. 4:**
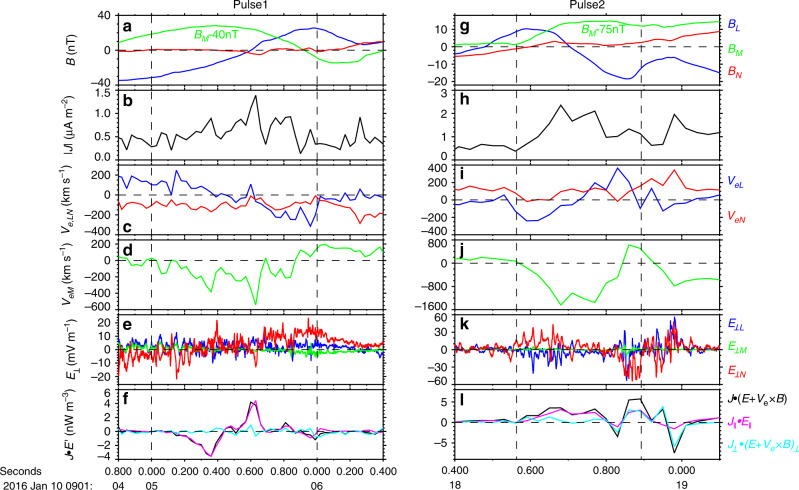
Direct evidence of the reconnection in Pulse1 and Pulse2. The data are shown in a local current (LMN) coordinate with *L* = (0.88, −0.47, −0.01)_GSE_, *M* = (0.25, 0.50, −0.83)_GSE_ and *N* = (0.39, 0.73, 0.56)_GSE_ for Pulse1, and *L* = (−0.92, 0.05, 0.39)_GSE_, *M* = (−0.18, 0.82, −0.55)_GSE_, and *N* = (−0.35, −0.57, −0.74)_GSE_ for Pulse2. **a**, **g** Magnetic field *B*_LMN_, with *B*_M_ shifted by −40 nT for Pulse1 and −75 nT for Pulse2. **b**, **h** The total current density intensity. **c**, **i** Electron velocity *V*_e,LN_. **d**, **j** Electron velocity *V*_eM_. **e**, **k** Perpendicular electric field *E*_⊥LMN_ in the spacecraft frame. The perpendicular electric fields mean electric field component perpendicular to the local magnetic field. **f**, **l***J* • *E*′ = *J* • (*E* + *V*_e_ × *B*).

We examined carefully each of the filamentary currents inside the varied magnetic field pulses and transferred the data of each varied magnetic field pulse into its local current (LMN) coordinates derived from the hybrid minimum variance analysis: *M* was determined as the direction of maximum current density in each varied magnetic field pulses; then *N* = *L*′ × *M*, where *L*′ is the direction of the maximum variance of the magnetic field; *L* = *M* × *N* (specific results of each varied magnetic field pulses are displayed in Supplementary Table 1). As a result, the signatures associated with reconnection were observed in all of the pulses. Especially, the bidirectional electron outflow jets, the related Hall electric field and significant energy conversion were simultaneously observed inside the four pulses at ~09:01:06, 09:01:16, 09:01:19, and 09:01:20 UT, marked by the arrow at the bottom of Fig. 2. The bidirectional plasma jets were the direct evidence of the ongoing reconnection. The two ongoing reconnection events dubbed Pulses 1 and 2 are exhibited in Fig. 4 in their individual local current layer coordinate. The other events can be found in the Supplementary Figs. 2 and 4.

There existed a few filamentary currents within the Pulse1 (Fig. 2c). We mainly focused on the first filamentary current at ~09:01:05.6 UT. The duration was about 1.0 s (~09:01:05.0 to ~09:01:06.0 UT). In order to accurately obtain its speed, we chose the magnetic signatures of the filamentary current with an evident time delay between any two of the four satellites. The timing method was finally performed to the points of *B*_L_ = 0 for this filamentary current and the speed was calculated to be 130.8 km s^−1^. So, the half-thickness of this filamentary current was estimated to be 65.4 km ~1.4 *d*_*i*_. Inside this filamentary current (Fig. 4a–f), *B*_L_ changed sign from negative to positive (Fig. 4a), *B*_M_ exhibited a bipolar variation with respect to the strong background field (~50 nT), and the current density peaked at the reversal point of *B*_L_ at 09:01:05.6 UT (Fig. 4b). It shows that the spacecraft crossed the thin filamentary current. In this crossing, the electron bulk flows in the *L* direction reversed from positive to negative with a speed up to 200 km s^−1^ ~4.1 *v*_A_ (blue trace, Fig. 4c) and *v*_eM_ got the minimum value just at the *v*_eL_ and *B*_L_ reversal point, *v*_eM_ ~ −500 km s^−1^ (Fig. 4d). Meanwhile, the perpendicular electric field in the normal direction of the current sheet *E*_⊥N_ changed sign also (red trace, Fig. 4e). It was basically negative in the positive *v*_eL_ and became positive in the negative *v*_eL_, consistent with the simulation for reconnection with a strong guide field^26^. At the center of the *v*_eL_ reversal point (09:01:05.6 UT, expected location of electron diffusion region), the energy dissipation *J* • *E*′ (Fig. 4f) was positive, up to 4 nW m^−3^. Thus, magnetic energy was releasing. At the two sides of the reconnection outflows, *J* • *E*′ was negative. This kind of the *J* • *E*′ distribution in the vicinity of the X-line is in good agreement with the simulation results^27^. Based on these observations, it is concluded that the spacecraft crossed the reconnection diffusion region from one outflow to the other, as illustrated in Fig. 1i. Immediately after the reconnection diffusion region, the spacecraft encountered another filamentary current at 09:01:06.3 UT, its half-thickness was estimated to be 0.14 *d*_i_, and reconnection signature was not observed there.

Figure 4g–l show one filamentary current at 09:01:18.7 UT inside the Pulse2. The duration of the current was ~0.32 s (~09:01:18.57 to ~09:01:18.89 UT) and the timing method was performed to the *B*_L_ maximum for this filamentary current. Finally, its speed and half-thickness were obtained to be 106.3 km s^−1^ and 17.0 km (~0.37 *d*_*i*_), respectively. The spacecraft crossed the filamentary current (Fig. 4g) and detected a strong guide field (*B*_M_ ~80 nT). In this crossing, the electron flow in the *L* direction reversed from negative (approximately −200 km s^−1^) to positive (approximately 300 km s^−1^, Fig. 4i) and −*v*_eM_ had the maximum value around the *v*_eL_ reversal (Fig. 4j). Meanwhile, the electric field component *E*_⊥N_ was positive in the negative *v*_eL_ and negative in the positive *v*_eL_. Furthermore, *J* • *E*′ was positive (~3 nW m^−3^) around the *v*_eM_ peak. Based on the analysis above, the reconnection with a strong guide field was indeed happening in this current and the schematic was displayed in Fig. 1j. The analogous reconnection events were detected also in the filamentary currents of pulses at ~09:01:16, and ~09:01:20 UT (see Supplementary Figs. 2 and 4).

Inside the rest pulses, although the bidirectional electron flows in the *L* direction were not observed, the reconnection outflow jets *v*_eL_, larger than the local Alfven speed, were always observed in some of the filamentary currents, as indicated by the arrows with round heads at the bottom of Fig. 2. It indicates that the reconnection was occurring but the spacecraft did not pass through the reconnection X-line region (see Supplementary Figs. 5–9). According to the observations, the reconnection was common in these varied magnetic field pulses and there was no ion-couple detected in these reconnection events, similar to the observations in the magnetosheath^28^ and in the magnetotail^29^. Moreover, the secondary reconnection was always with a strong guide field.

## Discussion

In the process of primary reconnection, magnetic energy is transferred from the large-scale current sheet into the ion-scale magnetic flux ropes. The energy dissipation via interaction between these flux ropes has been extensively studied^17,30,31^. Remarkably intense electric field structure^32–34^ and energy dissipation^35,36^ were detected inside flux ropes. However, it is confusing that why the energy dissipation can occur inside the simple helical magnetic structures. Focusing on the two big flux ropes embedded in the reconnection ion outflows, we find a few small-scale flux rope-like structures or the varied magnetic pulses inside each big flux rope. In all of these small-scale flux rope-like structures, the electron flow reversed at least at two directions, i.e., the parallel and one of the perpendicular directions. In some pulses, the electron flow reversed at all three directions. Namely, the electron vortices were three-dimensional. Furthermore, the electron vorticity was enhanced within each pulse.

It is well known that the electron flow shear can lead to excitation of the KH instability. The nonlinear evolution of the electron KH instability can lead to the vortices, which can explain the observed electron vortices inside the pulses here. The magnetic field component along the shear flow can be wrapped up with the evolving KH vortices and results in the compressed thin current layers where magnetic reconnection can be triggered^37–39^. The similar process was found in a large flux rope via a PIC simulation^40^, where the magnetic flux rope was bounded by two X-lines at the two ends, and thus the colliding reconnection outflows towards the center of the flux ropes were important to the electron dynamics. In our event reported here, there were no such colliding ion flows were observed.

In order to verify whether the KH instability can be excited here, we examined the instability criterion^41–43^ Δ*v*_eL_ > *v*_eA_/2, where Δ*v*_eL_ is the electron velocity variation across the filamentary currents and $v_{\rm{eA}}\,=\,B_{\mathrm{L}}/\sqrt {4\pi m_{\rm{e}}N}$ is the local electron Alfven speed. It is found that Δ*v*_eL_ was substantially smaller than *v*_eA_/2 around all of the filamentary currents (see Supplementary Figs. 1–9), i.e., the KH instability inside the big flux ropes was stable as the spacecraft passed through them. This discrepancy can be caused by the fact that the shear flow speed decreases as the KH instability evolves and can be very low during the nonlinear stage. Another scenario for occurrence of the observed varied magnetic field pulses is the secondary instability or the tearing-type instability^16^, driven by the strong magnetic shear across the electron current layer. As a result, secondary reconnection can occur in the filamentary currents. The formed flux rope was unstable to interchange instability as well driven by density gradients predicted by simulations^44^ and secondary reconnection was verified in the resulting narrow current layers. In our event, the intense density gradient was clear in both big flux ropes, consistent with the simulation results^44^. At present, we cannot distinguish which mechanism(s) is responsible for the generation of the varied magnetic field pulses.

According to our observation, it is undoubted that magnetic energy was released inside magnetic flux ropes via secondary reconnection. Given a number of the secondary reconnection inside the flux ropes, they may make a significant role on energy conversion during reconnection. More efforts are needed to devote the total energy budget in reconnection.

## Methods

### Local current coordinate system determination

To find secondary reconnection signatures, the spacecraft data should be examined in the local current coordinate system (LMN). A hybrid minimum variance analysis was used to determine the LMN coordinate system: *M* was along the direction of maximum current density in the filamentary current. *N* = *L*′ × *M*, where *L*′ was the direction of maximum variance of the magnetic field. *L* = *M* × *N*. The results are shown in Supplementary Table 1.

### Estimation of the filamentary currents speed

A multi‐spacecraft method was used to determine the speed of the filamentary currents. This method (timing method) is based on the time delay between the passages of the current sheet over four satellites. The relative position of the four MMS satellites can be found from MMS data. Then we chose the magnetic signatures of the filamentary currents with an evident time delay between any two of the four satellites. The timing method was performed to these signatures to obtain the speeds. The timing method was performed to the *B*_L_ maximum for the events E3, E5, E7, E8, and E9, to the *B*_L_ minimum for the events E2 and E6, and to the points of *B*_L_ = 0 for the events E1 and E4. The results are shown in Supplementary Table 2.

## Supplementary information

Supplementary Information

## Data Availability

All the MMS data used in this work are available at the MMS data center (https://lasp.colorado.edu/mms/sdc/public/). Magnetic field and electric field data are available at https://lasp.colorado.edu/mms/sdc/public/datasets/fields/. The electron and ion moment data can be found at https://lasp.colorado.edu/mms/sdc/public/datasets/fpi/.

## Code availability

All the spacecraft data in this study are collected and analyzed with the IDL software (spedas) available to the public in the MMS database website. The IDL spedas package is available at https://lasp.colorado.edu/mms/sdc/public/software/.

## References

[1] Chen LJ (2008). Observation of energetic electrons within magnetic islands. Nat. Phys..

[2] Sibeck DG (1984). Magnetotail flux ropes. Geophys. Res. Lett..

[3] Olson J (2016). Experimental demonstration of the collisionless plasmoid instability below the ion kinetic scale during magnetic reconnection. Phys. Rev. Lett..

[4] Slavin JA (2003). Geotail observations of magnetic flux ropes in the plasma sheet. J. Geophys. Res..

[5] Drake JF, Swisdak M, Che H, Shay MA (2006). Electron acceleration from contracting magnetic islands during reconnection. Nature.

[6] Du SB, Guo F, Zank GP, Li XC, Stanier A (2018). Plasma energization in colliding magnetic flux ropes. Astrophys. J..

[7] Fu XR, Lu QM, Wang S (2006). The process of electron acceleration during collisionless magnetic reconnection. Phys. Plasmas.

[8] Oka M, Phan TD, Krucker S, Fujimoto M, Shinohara I (2010). Electron acceleration by multi-island coalescence. Astrophys. J..

[9] Wang RS, Lu QM, Du AM, Wang S (2010). In situ observations of a secondary magnetic island in an ion diffusion region and associated energetic electrons. Phys. Rev. Lett..

[10] Daughton W, Scudder J, Karimabadi H (2006). Fully kinetic simulations of undriven magnetic reconnection with open boundary conditions. Phys. Plasmas.

[11] Karimabadi H, Daughton W, Scudder J (2007). Multi-scale structure of the electron diffusion region. Geophys. Res. Lett..

[12] Paschmann G (1982). Plasma and magnetic field characteristics of magnetic flux transfer events. J. Geophys. Res..

[13] Russell CT, Elphic RC (1978). Initial ISEE magnetometer results: magnetopause observations. Space Sci. Rev..

[14] Pu ZY (2013). Magnetic topologies of an in vivo FTE observed by Double Star/TC-1 at Earth’s magnetopause. Geophys. Res. Lett..

[15] Lee LC, Fu ZF (1985). A theory of magnetic flux transfer at the Earth’s magnetopause. Geophys. Res. Lett..

[16] Daughton W (2011). Role of electron physics in the development of turbulent magnetic reconnection in collisionless plasmas. Nat. Phys..

[17] Wang RS (2016). Coalescence of magnetic flux ropes in the ion diffusion region of magnetic reconnection. Nat. Phys..

[18] Burch JL, Moore TE, Torbert RB, Giles BL (2016). Magnetospheric multiscale overview and science objectives. Space Sci. Rev..

[19] Eastwood JP (2016). Ion-scale secondary flux ropes generated by magnetopause reconnection as resolved by MMS. Geophys. Res. Lett..

[20] Wang RS (2017). Interaction of magnetic flux ropes via magnetic reconnection observed at the magnetopause. J. Geophys. Res..

[21] Wang SM (2019). Anisotropic electron distributions and whistler waves in a series of the flux transfer events at the magnetopause. J. Geophys. Res..

[22] Schwartz SJ (1998). Shock and discontinuity normals, Mach numbers, and related parameters. ISSI Sci. Rep. Ser..

[23] Zenitani S, Hesse M, Klimas A, Kuznetsova M (2011). New measure of the dissipation region in collisionless magnetic reconnection. Phys. Rev. Lett..

[24] Wan M (2016). Intermittency, coherent structures and dissipation in plasma turbulence. Phys. Plasmas.

[25] Phan TD (2016). MMS observations of electron-scale filamentary currents in the reconnection exhaust and near the X line. Geophys. Res. Lett..

[26] Oieroset M (2016). MMS observations of large guide field symmetric reconnection between colliding reconnection jets at the center of a magnetic flux rope at the magnetopause. Geophys. Res. Lett..

[27] Zenitani S, Hesse M, Klimas A, Black C, Kuznetsova M (2011). The inner structure of collisionless magnetic reconnection: the electron-frame dissipation measure and Hall fields. Phys. Plasmas.

[28] Phan TD (2018). Electron magnetic reconnection without ion coupling in Earth’s turbulent magnetosheath. Nature.

[29] Wang RS (2018). An electron-scale current sheet without bursty reconnection signatures observed in the near-Earth tail. Geophys. Res. Lett..

[30] Pritchett PL (2007). Kinetic properties of magnetic merging in the coalescence process. Phys. Plasmas.

[31] Zhou M (2017). Coalescence of macroscopic flux ropes at the subsolar magnetopause: magnetospheric multiscale observations. Phys. Rev. Lett..

[32] Hwang KJ (2018). Small-scale flux transfer events formed in the reconnection exhaust region between two X lines. J. Geophys. Res..

[33] Stawarz JE (2018). Intense electric fields and electron-scale substructure within magnetotail flux ropes as revealed by the magnetospheric multiscale mission. Geophys. Res. Lett..

[34] Wang RS (2016). Electrostatic and electromagnetic fluctuations detected inside magnetic flux ropes during magnetic reconnection. J. Geophys. Res..

[35] Fu HS (2017). Intermittent energy dissipation by turbulent reconnection. Geophys. Res. Lett..

[36] Huang SY (2019). Observations of flux ropes with strong energy dissipation in the magnetotail. Geophys. Res. Lett..

[37] Nakamura TKM, Daughton W, Karimabadi H, Eriksson S (2013). Three-dimensional dynamics of vortex-induced reconnection and comparison with THEMIS observations. J. Geophys. Res..

[38] Nakamura TKM, Hasegawa H, Shinohara I, Fujimoto M (2011). Evolution of an MHD-scale Kelvin-Helmholtz vortex accompanied by magnetic reconnection: two-dimensional particle simulations. J. Geophys. Res..

[39] Nykyri K, Otto A (2001). Plasma transport at the magnetospheric boundary due to reconnection in Kelvin-Helmholtz vortices. Geophys. Res. Lett..

[40] Huang C (2017). Development of turbulent magnetic reconnection in a magnetic island. Astrophys. J..

[41] Fermo RL, Drake JF, Swisdak M (2012). Secondary magnetic islands generated by the Kelvin-Helmholtz instability in a reconnecting current sheet. Phys. Rev. Lett..

[42] Huang C (2015). Magnetic islands formed due to the Kelvin-Helmholtz instability in the outflow region of collisionless magnetic reconnection. Geophys. Res. Lett..

[43] Zhong ZH (2018). Evidence for secondary flux rope generated by the electron Kelvin-Helmholtz instability in a magnetic reconnection diffusion region. Phys. Rev. Lett..

[44] Lapenta G, Markidis S, Goldman MV, Newman DL (2015). Secondary reconnection sites in reconnection-generated flux ropes and reconnection fronts. Nat. Phys..

