# Supplementary material for: Direct evidence of secondary reconnection inside filamentary currents of magnetic flux ropes during magnetic reconnection
Source: Nat Commun. 2020 Aug 7;11:3964. doi: 10.1038/s41467-020-17803-3 (PMC7415135; doi:10.1038/s41467-020-17803-3)
Supplement: Supplementary file 1 — Supplementary Information [file 41467_2020_17803_MOESM1_ESM.pdf]

Supplementary Information

Direct evidence of secondary reconnection inside filamentary currents of magnetic flux ropes during magnetic reconnection

Wang et al.

Events	Time	L (GSE)	M (GSE)	N (GSE)	Crossing
E1	09:01:05.0~09:01:06.0	(0.88,-0.47,-0.01)	(0.25,0.50,-0.83)	(0.39,0.73,0.56)	X-line
E2	09:01:15.9~09:01:16.2	(0.69,0.71,0.13)	(-0.61,0.67,-0.43)	(-0.40,0.22,0.89)	X-line
E3	09:01:18.57~09:01:18.89	(-0.92,0.05,0.39)	(-0.18,0.82,-0.55)	(-0.35,-0.57,-0.74)	X-line
E4	09:01:19.2~09:01:20.1	(-0.70,0.69,0.16)	(0.38,0.56,-0.73)	(-0.60,-0.45,-0.66)	X-line
E5	09:01:02.1-09:01:02.7	(-0.29,0.74,0.61)	(-0.34,-0.67,0.66)	(0.89,-0.02,0.45)	exhaust
E6	09:01:03.43~09:01:03.68	(0.96,-0.16,0.23)	(0.26,0.81,-0.53)	(-0.10,0.57,0.82)	exhaust
E7	09:01:04.5~09:01:04.7	(-0.87,-0.44,-0.22)	(-0.26,0.79,-0.56)	(0.42,-0.43,-0.80)	exhaust
E8	09:01:17.2~09:01:17.9	(0.99,0.07,0.08)	(-0.04,0.96,-0.28)	(-0.10,0.27,0.96)	exhaust
E9	09:01:21.6~09:01:22.5	(-0.19,-0.89,0.41)	(-0.67,0.42,0.61)	(-0.72,-0.16,-0.68)	exhaust

Supplementary Table 1. The time intervals and local current coordinate systems for all of the identified reconnection events.

Events	Time	Velocity (km/s)	Duration (s)	half-thickness (d_i)
E1	09:01:05.0~09:01:06.0	130.8*(0.062,0.004,-0.998)$_{LMN}$	1.0	1.4
E2	09:01:15.9~09:01:16.2	82.3*(-0.083,-0.145,-0.986)$_{LMN}$	0.3	0.3
E3	09:01:18.57~09:01:18.89	106.3*(-0.286,0.013,0.958)$_{LMN}$	0.32	0.4
E4	09:01:19.2~09:01:20.1	129.0*(-0.020,-0.483,-0.876)$_{LMN}$	0.9	1.3
E5	09:01:02.1-09:01:02.7	114.2*(0.155,-0.203,0.967)$_{LMN}$	0.6	0.8
E6	09:01:03.43~09:01:03.68	124.4*(0.149,-0.307,-0.940)$_{LMN}$	0.25	0.3
E7	09:01:04.5~09:01:04.7	111.7*(-0.159,-0.039,0.987)$_{LMN}$	0.2	0.2
E8	09:01:17.2~09:01:17.9	139.1*(-0.125,-0.273,-0.954)$_{LMN}$	0.7	1.1
E9	09:01:21.6~09:01:22.5	97.1*(-0.140,0.006,-0.990)$_{LMN}$	0.9	1.0

Supplementary Table 2. The velocities and durations for all of the identified reconnection events.

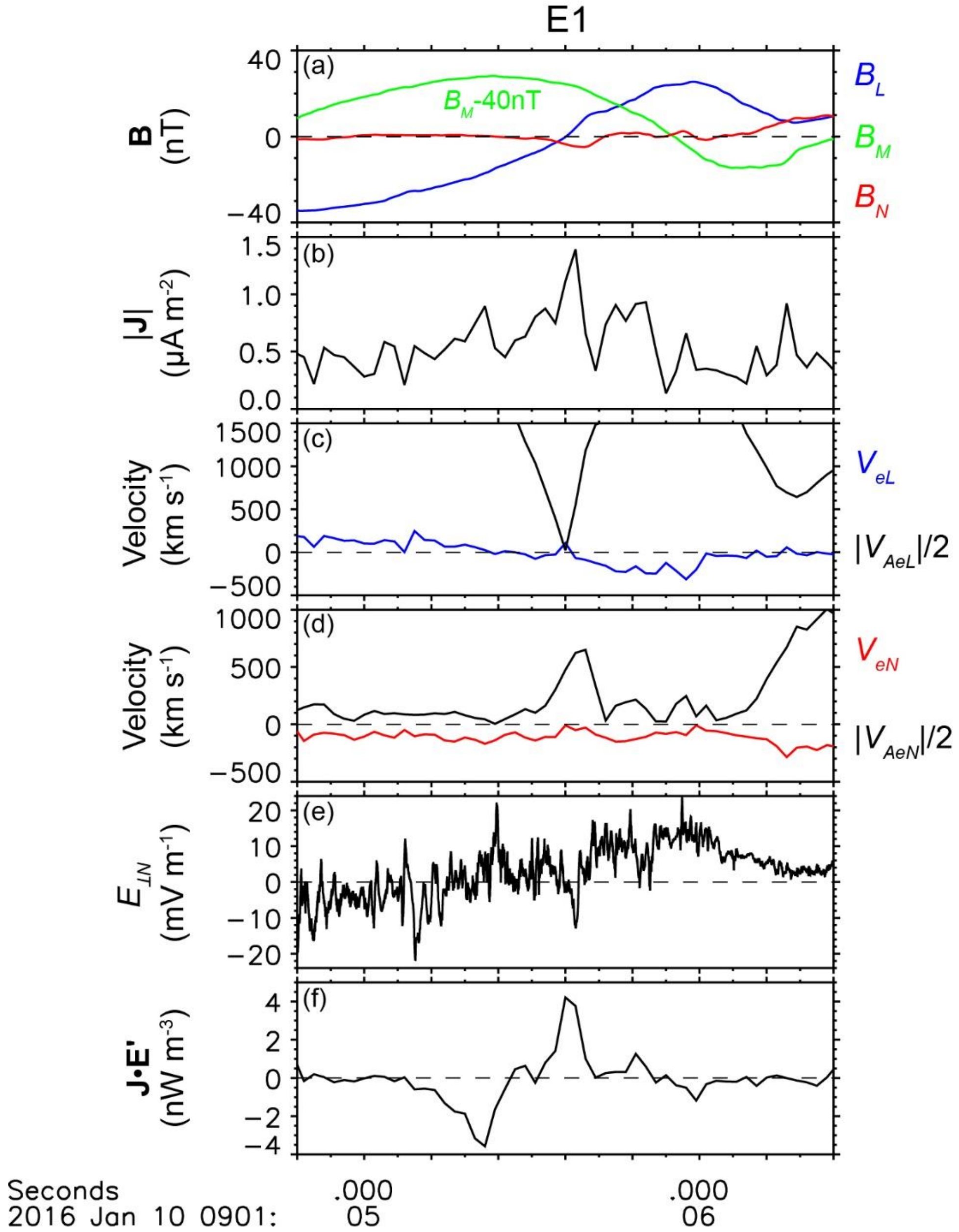

Supplementary Figure 1| Observations of the secondary reconnection sites E1. (a), magnetic field B_{LMN}. **(b),** the total current density intensity. **(c),** electron bulk velocity and the half local electron Alfven speed along the $\mathbf{L}$ direction. **(d),** electron bulk velocity and the half local electron Alfven speed along the $\mathbf{N}$ direction. **(e),** perpendicular electric field in the $\mathbf{N}$ direction. **(f),** $\mathbf{J}\cdot\mathbf{E}' = \mathbf{J}\cdot\left(\mathbf{E}+\mathbf{V_e}\times\mathbf{B}\right)$.

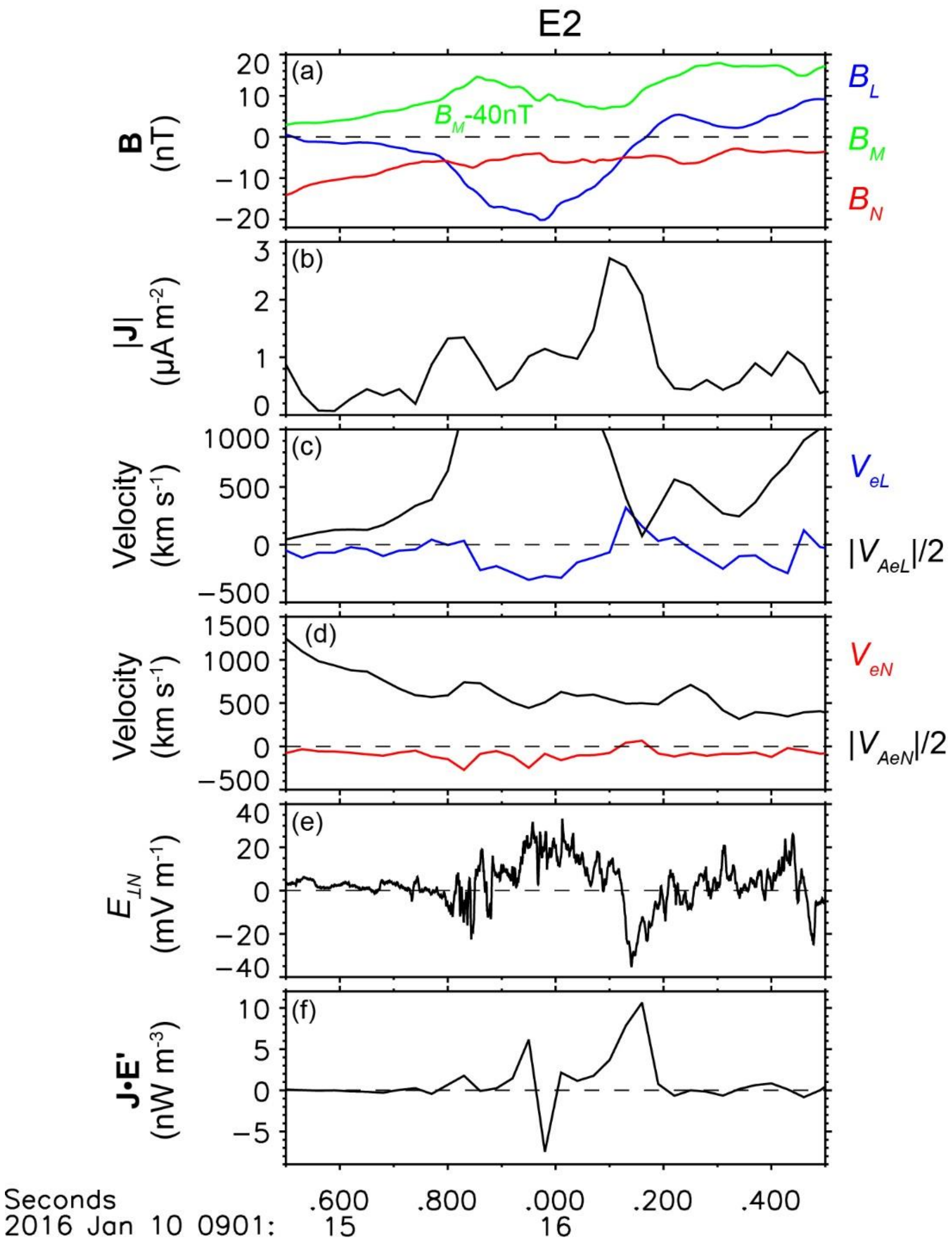

Supplementary Figure 2| Observations of the secondary reconnection sites E2. (a)-(f), the data are shown in the same format as in Supplementary Figure 1.

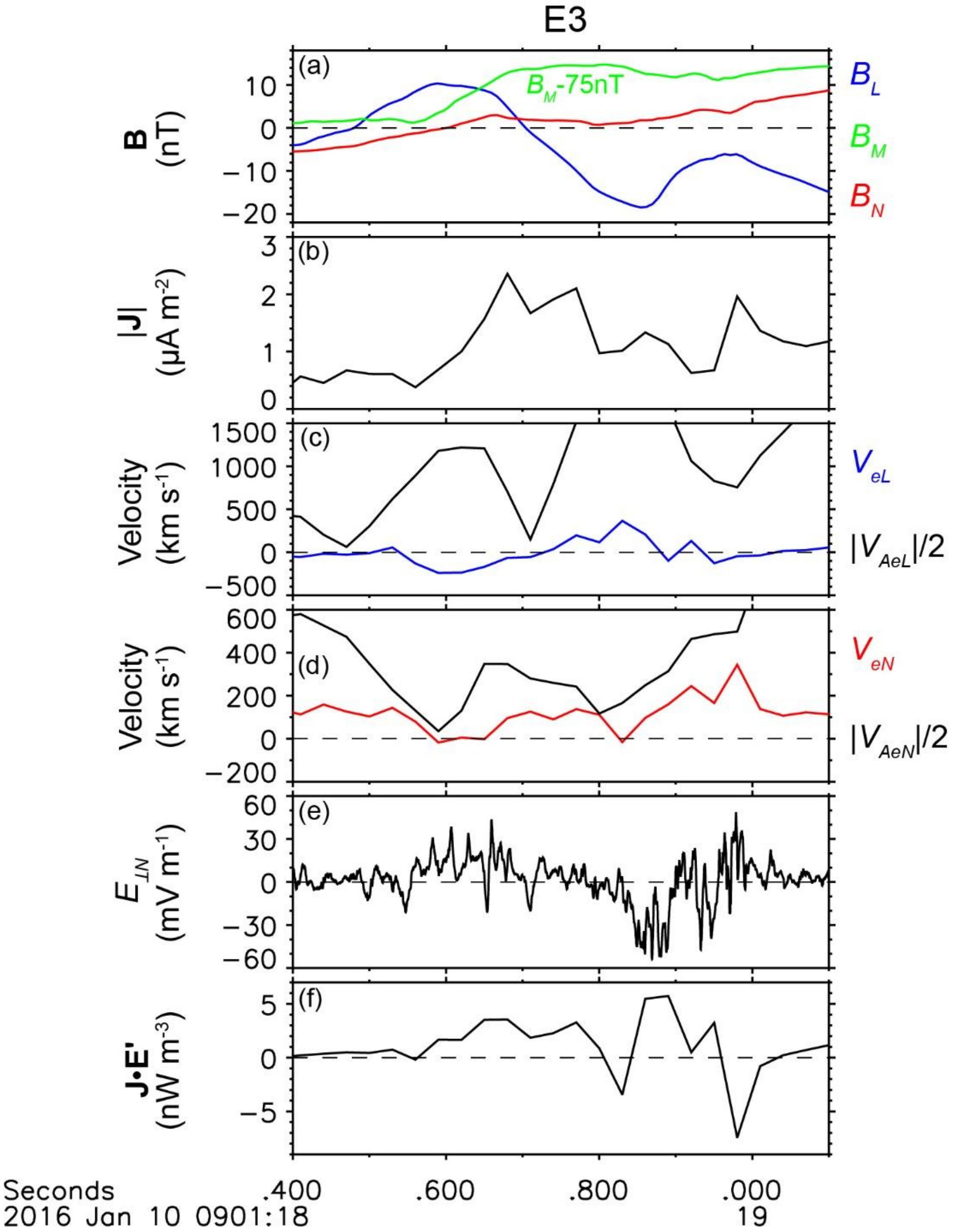

Supplementary Figure 3| Observations of the secondary reconnection sites E3. (a)-(f), the data are shown in the same format as in Supplementary Figure 1.

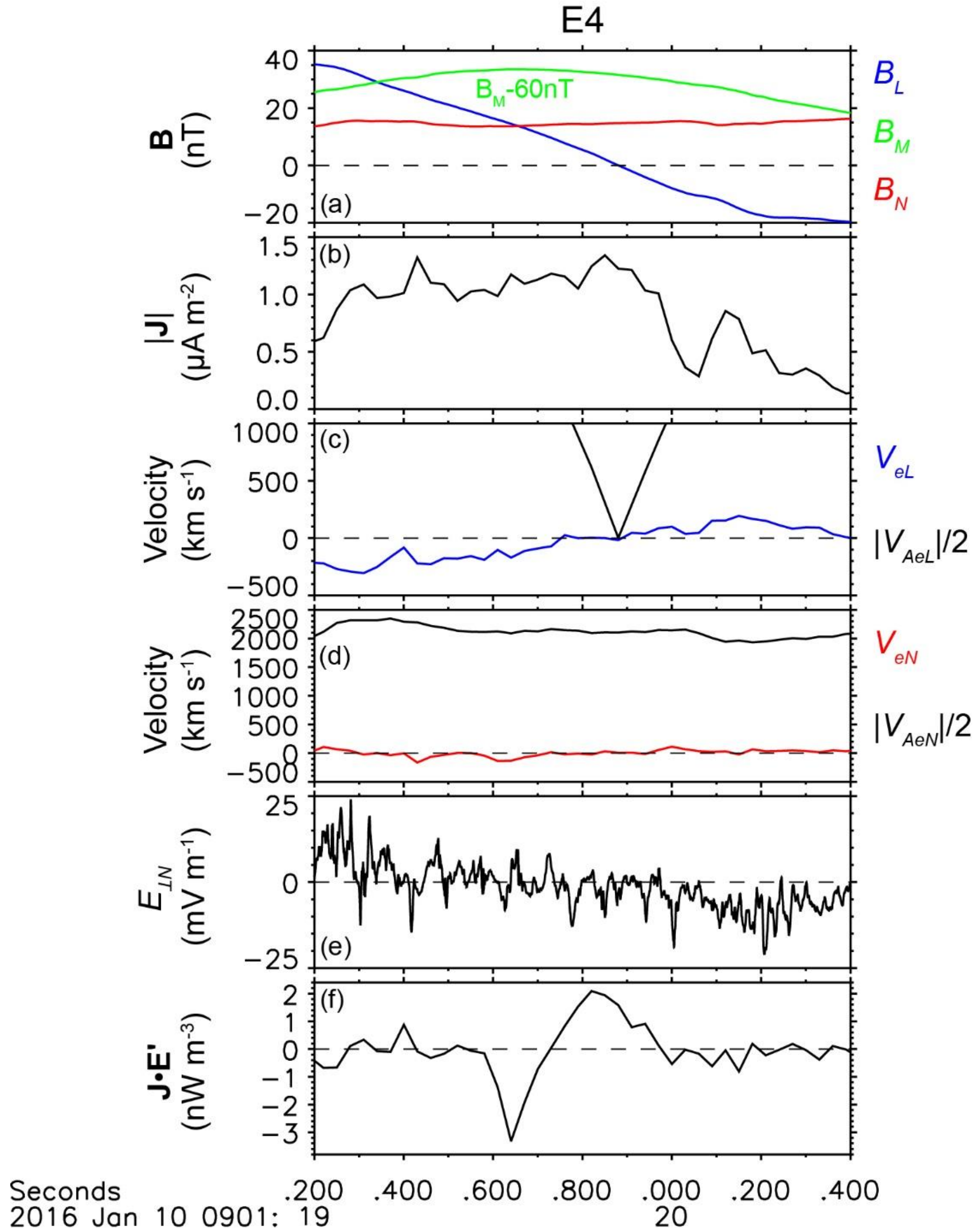

Supplementary Figure 4| Observations of the secondary reconnection sites E4. (a)-(f), the data are shown in the same format as in Supplementary Figure 1.

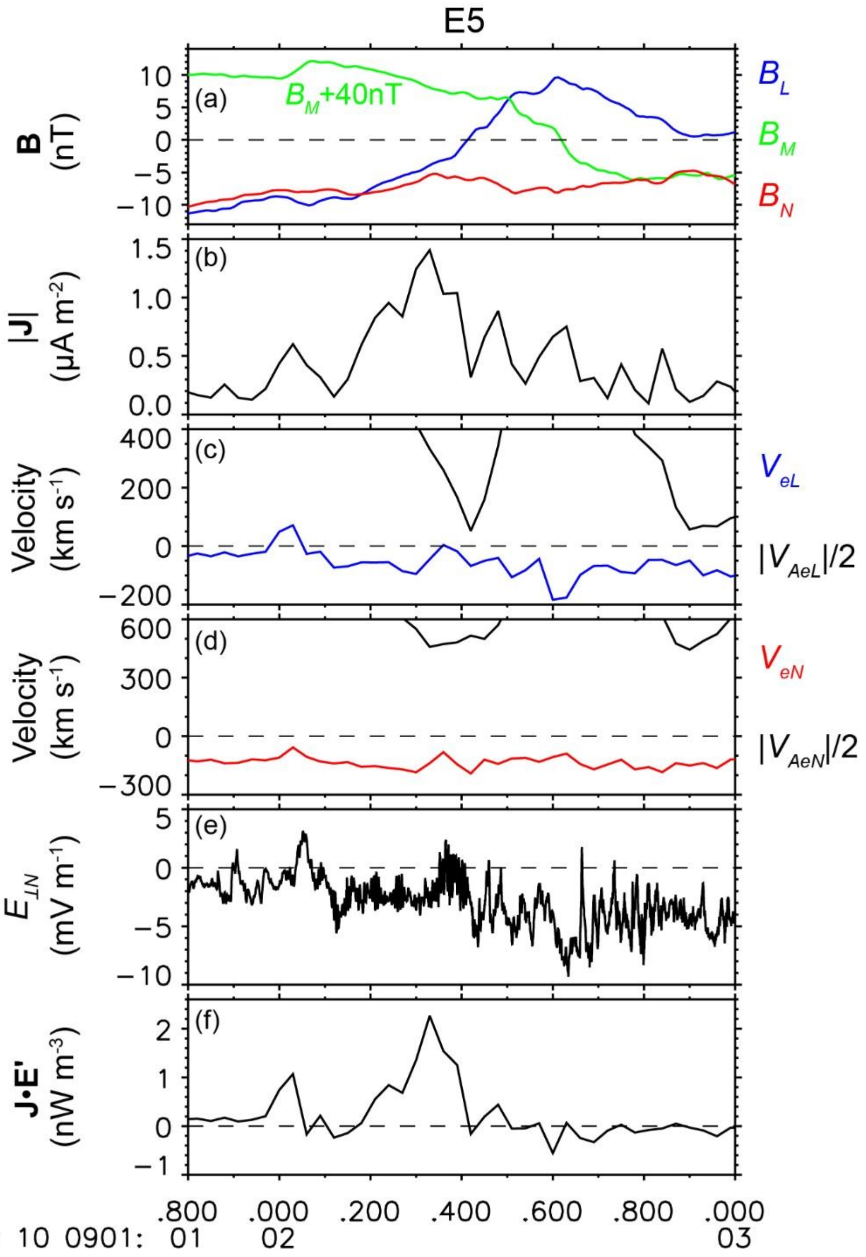

Supplementary Figure 5| Observations of the secondary reconnection exhaust E5.

(a)-(f), the data are shown in the same format as in Supplementary Figure 1.

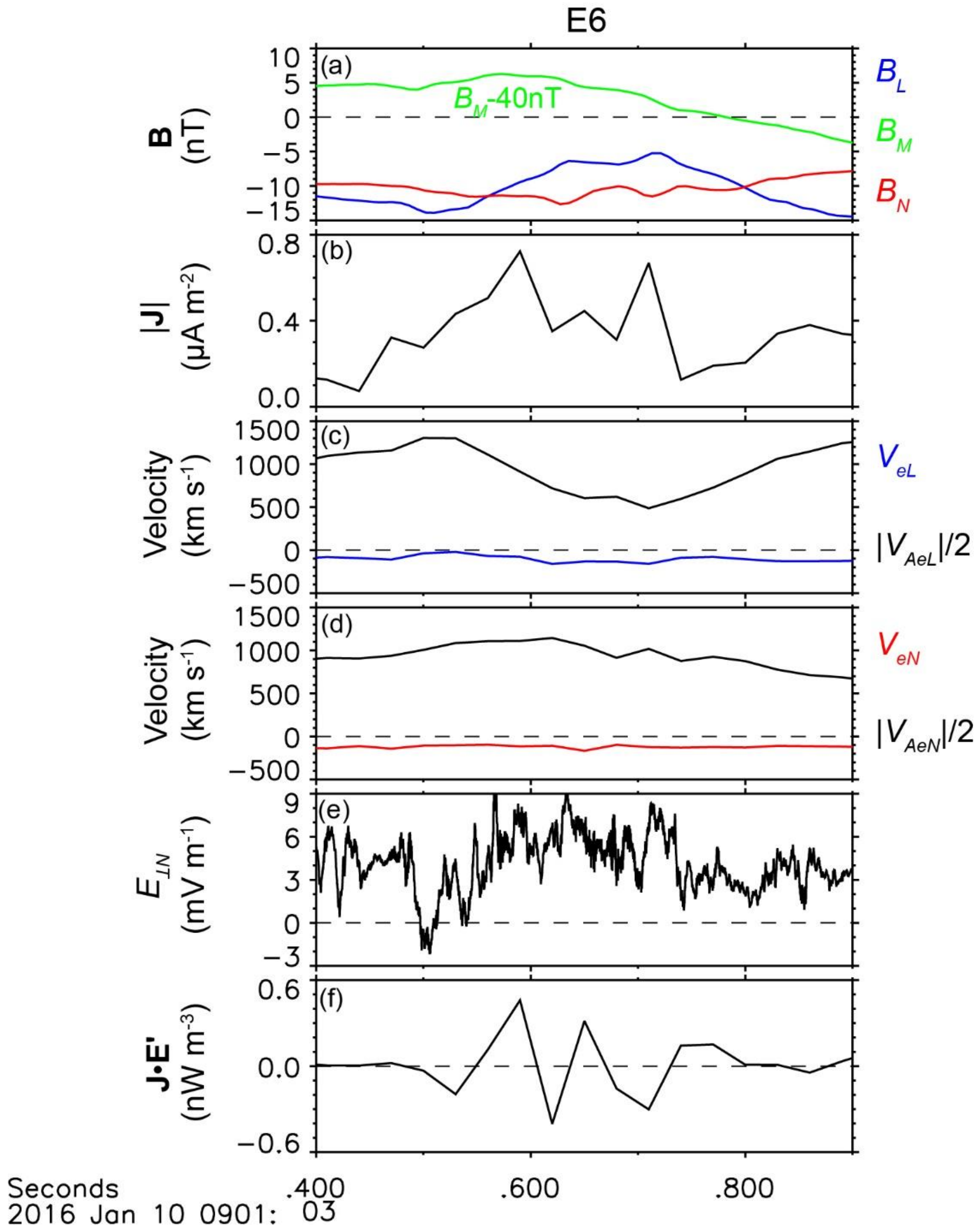

Supplementary Figure 6| Observations of the secondary reconnection exhaust E6.

(a)-(f), the data are shown in the same format as in Supplementary Figure 1.

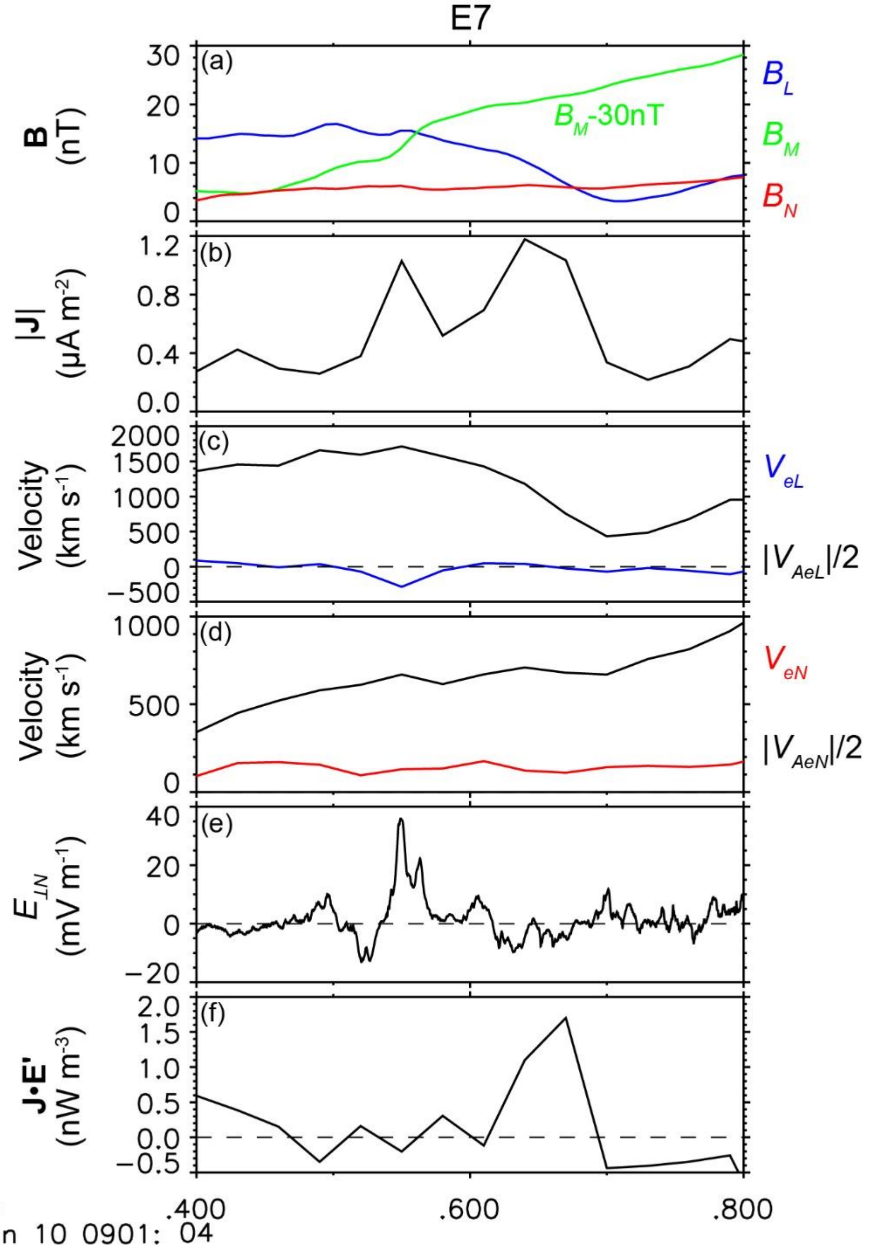

Supplementary Figure 7| Observations of the secondary reconnection exhaust E7.

(a)-(f), the data are shown in the same format as in Supplementary Figure 1.

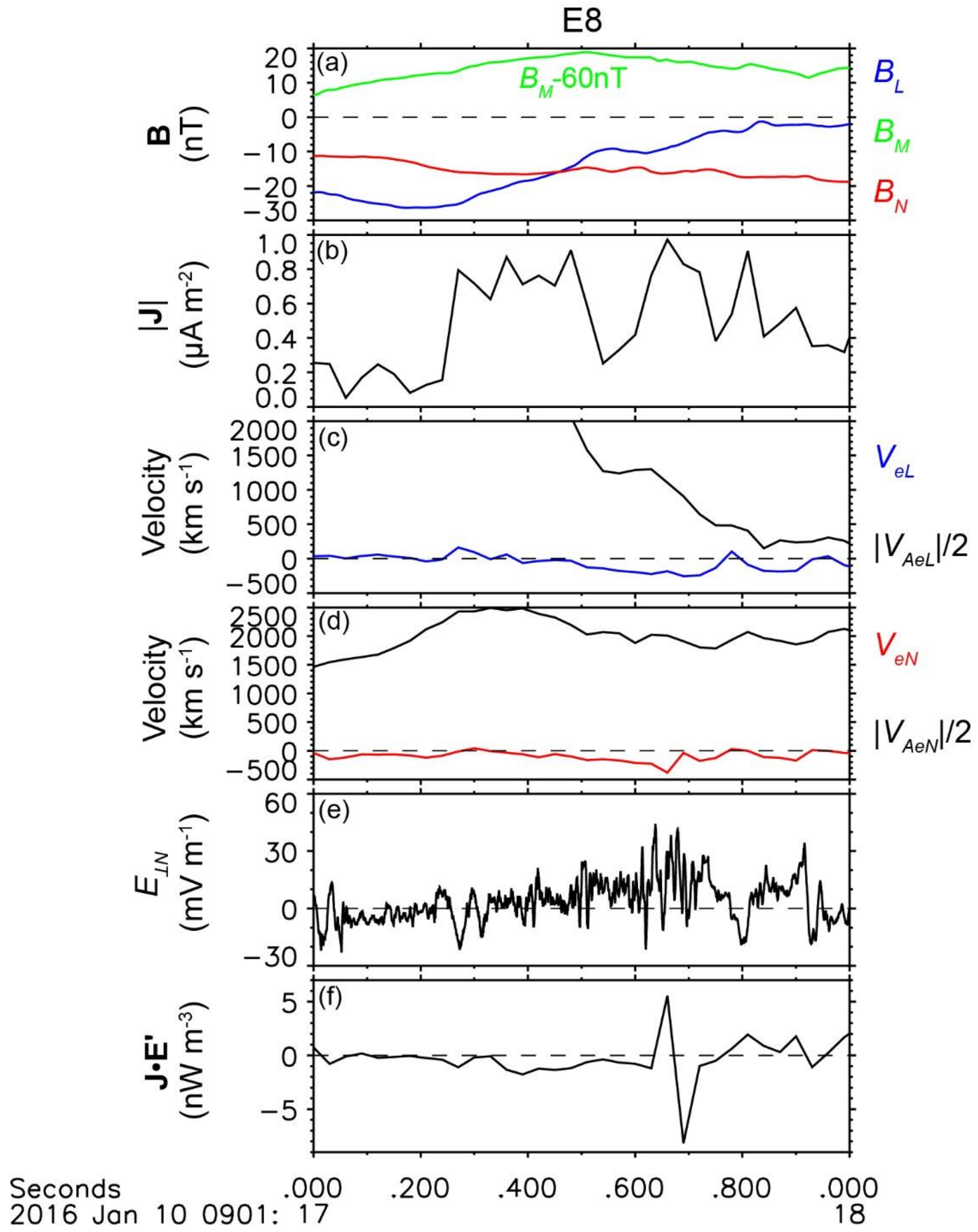

Supplementary Figure 8| Observations of the secondary reconnection exhaust E8.

(a)-(f), the data are shown in the same format as in Supplementary Figure 1.

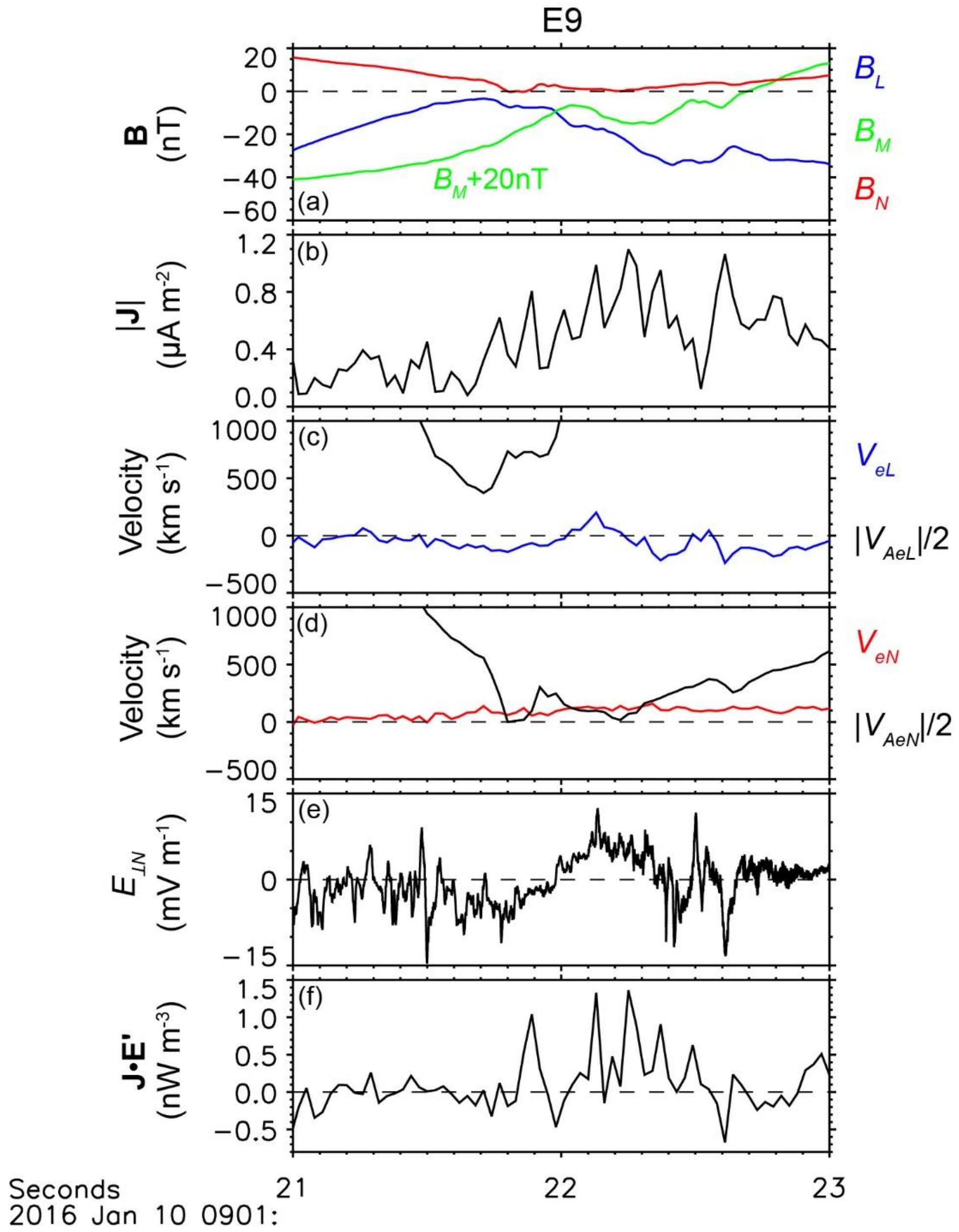

Supplementary Figure 9| Observations of the secondary reconnection exhaust E9.

(a)-(f), the data are shown in the same format as in Supplementary Figure 1.
